# A quality improvement (QI) project to ensure females on valproate in a CMHT outpatient clinic, eput are registered on the valproate pregnancy prevention programme

**DOI:** 10.1192/bjo.2021.502

**Published:** 2021-06-18

**Authors:** Adebayo Emmanuel, Parvathy Pillay, Vyasa Immadisetty

**Affiliations:** 1 ST5 Essex Partnership University NHS Foundation Trust; 2 Consultant Psychiatrist Essex Partnership Unniversity NHS Foundation Trust

## Abstract

**Aims:**

Recent MHRA guidelines, state that Valproate medicines must no longer be used in women or girls of childbearing potential due to its highly teratogenic effects unless a Pregnancy Prevention Programme is in place. We carried out a service evaluation to determine if there was any way of identifying such patients with the aim of setting one up if required.

**Background:**

Valproate is highly teratogenic and evidence supports that use in pregnancy leads to physical birth defects in 10 in every 100 babies (compared with a background rate of 2 to 3 in 100) and neurodevelopmental disorders in approximately 30 to 40 in every 100 children born to mothers taking Valproate. Data from a previous inpatient audit identified 35 females of childbearing age and none was on a pregnancy prevention plan. Audits done thereafter confirmed there was no system available for identifying such patients. Availability and accessibility to a synchronised IT system, which alerts when the yearly review is due is consistently identified as a contributory factor.

**Method:**

A request via the Medicines management team was sent to the GP surgeries within the catchment area to assist in identifying female patients on their records on Valproate registered on the Pregnancy Prevention Programme.

The Plan-Do-Study-Act (PDSA) Quality Improvement (QI) model was used to bring about change. The target set to achieve was a 50% reduction in the prescriptions of Valproate in such patient groups.

**Result:**

We had 10 out of the 50 GP surgeries contacted responded with a list of female patients on Valproate, a total of 25 patients’ altogether. In total, 4 patients out of the 25 were registered on the Pregnancy Prevention Programme and the overall non-compliance rate was 86%. . Factors believed to contribute to the low numbers include a lack of a system for registering women of childbearing age on the pregnancy protection plan and the recent introduction of GDPR regulation.

**Conclusion:**

There are ongoing discussions with various stakeholders like the Medicines management team, Pharmacists, electronic records team (IT) and other clinicians regarding inserting an alert in the electronic system that reminds clinicians to register all such women on the Pregnancy Prevention Programme, while automatically creating a yearly reminder for completion of the annual risk acknowledgement form.

